# Low prevalence of ideal cardiovascular health in Peru

**DOI:** 10.1136/heartjnl-2017-312255

**Published:** 2018-01-11

**Authors:** Catherine P Benziger, José Alfredo Zavala-Loayza, Antonio Bernabe-Ortiz, Robert H Gilman, William Checkley, Liam Smeeth, German Malaga, Juan Jaime Miranda, CRONICAS Cohort Study group

**Affiliations:** 1 CRONICAS Center of Excellence in Chronic Diseases, Universidad Peruana Cayetano Heredia, Lima, Peru; 2 Division of Cardiology, University of Washington, Seattle, WA, USA; 3 Faculty of Epidemiology and Population Health, London School of Hygiene andTropical Medicine, London, UK; 4 Department of International Health, Johns Hopkins University, Baltimore, MD, USA; 5 Research Division, Asociación Benéfica PRISMA, Lima, Peru; 6 Division of Pulmonary and Critical Care, School of Medicine Johns Hopkins University, Baltimore, MD, USA; 7 Facultad de Medicina “Alberto Hurtado”, Universidad Peruana Cayetano Heredia, Lima, Peru

**Keywords:** cardiac risk factors and prevention, obesity, epidemiology, global disease patterns

## Abstract

**Background:**

The prevalence of and factors associated with ideal cardiovascular health (ICH) by sociodemographic characteristics in Peru is not well known.

**Methods:**

The American Heart Association’s ICH score comprised 3 ideal health factors (blood pressure, untreated total cholesterol and glucose) and 4 ideal health behaviours (smoking, body mass index, high physical activity and fruit and vegetable consumption). ICH was having 5 to 7 of the ideal health metrics. Baseline data from the Center of Excellence in Chronic Diseases, a prospective cohort study in adults aged ≥35 years in 4 Peruvian settings, was used (n=3058).

**Results:**

No one met all 7 of ICH metrics while 322 (10.5%) had ≤1 metric. Fasting plasma glucose was the most prevalent health factor (72%). Overall, compared with ages 35–44 years, the 55–64 years age group was associated with a lower prevalence of ICH (prevalence ratio 0.54, 95% CI 0.40 to 0.74, P<0.001). Compared with those in the lowest tertile of socioeconomic status, those in the middle and highest tertiles were less likely to have ICH after adjusting for sex, age and education (P<0.001).

**Conclusion:**

There is a low prevalence of ICH. This is a benchmark for the prevalence of ICH factors and behaviours in a resource-poor setting.

## Introduction

Ideal, intermediate and poor cardiovascular health was defined by the American Heart Association (AHA) 2020 Impact goals and has shifted the focus globally on promotion of health and control of risk rather than solely on prevention and treatment of disease.^1 2^ Ideal health is the simultaneous presence of three ideal health factors (normal cholesterol, normal blood pressure and no diabetes) and four ideal health behaviours (non-smoking, normal weight, high physical activity and ideal diet).^2^ Prior studies suggest that individuals with 5, 6 or 7 ideal cardiovascular health (ICH) metrics have up to 10 times lower levels of ischaemic heart disease, cardiovascular disease (CVD) mortality, stroke^3^ and all-cause mortality compared with those with 0 to 1 ICH metric.^4–7^ Prevalence and outcome estimates of ICH metrics in Latin America are lacking.^4^ Peru offers an opportunity to assess the impact of geographical variation on cardiovascular risk by combining settings on the spectrum of both rural-urban development as well as lowland-highland scenarios. Disparities in cardiovascular risk factors have been described by age, sex, ethnicity and geographical region in other populations,^5–14^ but limited data exist in Latin America.^11 13^

The objective was to determine the prevalence of ideal, intermediate and poor cardiovascular health in a Latin American population, as well as calculate the mean cardiovascular health score by sociodemographic characteristics in Peru.

## Methods

### Study design, setting and participants

Design of the Center of Excellence in Chronic Diseases (CRONICAS) study were reported previously.^15^ CRONICAS is a prospective age-stratified and sex-stratified cohort study in adults aged ≥35 years in four Peruvian settings: Lima (Peru’s capital, costal urban, highly urbanised), urban and rural Puno (both high-altitude) and Tumbes (costal semirural). Randomly selected participants by sex and age groups (aged 35–44, 45–54, 55–64 and 65+ years) were taken from census data at each study site. Data from baseline (collected in 2010) were used. Exclusion criteria included pregnant women, those unable to provide informed consent or respond to the questionnaires and bedridden individuals.

### Data collection

A team of community health workers was trained to enrol participants and to conduct the questionnaires assessing sociodemographic and behavioural factors. Sociodemographic variables included sex, age groups, study site, education level and socioeconomic status (an aggregation of assets and household facilities into a wealth index and divided in tertiles^16^).

### American Heart Association definition of ICH

ICH was defined as the simultaneous presence of ideal health factors (untreated blood pressure <120/<80 mm Hg, untreated total cholesterol (TC) <200 mg/dL and untreated fasting plasma glucose <100 mg/dL) and ideal health behaviours (never smoker, body mass index (BMI) <25 kg/m^2^, moderate physical activity ≥150 min/week and ideal diet with four of the five components of the Dietary Approach to Stop Hypertension (DASH) diet).^17^ ICH was described in two ways: (1) percentage of the population with ICH (5–7 ideal metrics) and (2) mean healthy lifestyle score (range from 0 to 7). Intermediate and poor cardiovascular health were also assessed (3–4, and 0–2 ideal metrics, respectively). Participants self-reported their health in the question, "Would you consider that your health is: very good, good, regular, bad and very bad". The perception of health was also compared with the ICH score.

### Assessment of ICH factors

Participants were invited to a clinic visit where systolic (SBP) and diastolic (DBP) blood pressure were measured in triplicate with the mean of the last two measurements used for the analysis.^15^ Total cholesterol, triglycerides and high-density lipoprotein cholesterol (HDL-c) were measured in serum, whereas fasting glucose was assessed in plasma and samples were obtained and sent to central laboratories for processing.^1 15^

Ideal blood pressure was defined as blood pressure <120/<80 mm Hg and without any antihypertensive medication, intermediate was SBP 120–139 or DBP 80–89 mm Hg or treated to blood pressure <120/<80 mm Hg, and poor was blood pressure ≥140/≥90 mm Hg. Ideal total cholesterol was TC <200 mg/dL and without any cholesterol-lowering medication, intermediate was TC 200–239 mg/dL or treated to TC <200 mg/dL and poor was TC ≥240 mg/dL. Ideal fasting blood glucose was <100 mg/dL and without any glucose-lowering medication, intermediate was glucose 100–125 mg/dL or treated to <100 mg/dL and poor was glucose ≥126 mg/dL.

### Assessment of ICH behaviours

BMI (kg/m^2^) was computed from weight and standing height, measured in the clinic setting.^15^ Ideal BMI was defined as 18.5–24.9 kg/m^2^ and poor was BMI ≥30 kg/m^2^.

Smoking was defined as ideal if self-report of never having smoked or a former smoker who quit >12 months ago. Intermediate smoking included those who quit within the past 1–12 months, and poor included daily smoker (>1 cigarette/day or if former but last cigarette was in the past 1 month).

For dietary intake, a food questionnaire was used that assessed dietary patterns over the past month.^15^ Healthy diet score used to define ICH included fruits and vegetables ≥4.5 cups/day, fish ≥2 3.5 oz. /week, whole grains ≥3 1 oz. servings/day, sodium <1500 mg/day, added sugar in sugar-sweetened beverages <450 kcal/week. Since only 89 (3%) participants met at least four of the five components of the diet, we used intake of fruits and vegetables ≥4.5 times/day as a surrogate of ideal diet in the definition of ICH, as used in prior studies.^9 18^ For this variable, discrimination between intermediate and poor nutrition status was not possible as it was dichotomised to ideal (≥4.5 times/day) and poor (<4.5 times/day).

Physical activity was based on both leisure time and transportation-based domain of International Physical Activity Questionnaire as recommended for Latin American populations.^19^ Ideal physical activity was ≥150 min/week moderate or ≥75 min/week vigorous or ≥150 min/week moderate and vigorous activity. Poor physical activity was no physical activity reported with intermediate activity as any other amounts of activity.

### Definition of treatment control for ICH factors

Use of antihypertensive, cholesterol-lowering and glucose-lowering medications within the past month of interview was self-reported and the participant was categorised as either intermediate cardiovascular health or poor cardiovascular health based on their clinical or laboratory measurements for each metric and the threshold definitions for ideal control were stated above, in agreement with current clinical practice guidelines.^19 20^ Poor control was defined as blood pressure ≥140/≥90 mm Hg, total cholesterol ≥240 mg/dL and glucose ≥126 mg/dL. Participants self-reported history of heart disease or stroke by a physician.

### Statistical analysis

A description of the socio-demographic, behavioural and clinical variables overall and according to sex was performed. Participants that did not have all of the cardiometabolic factors measured were excluded from the analysis (n=504; primary reason for exclusion: no blood pressure recorded n=387 or incomplete diet questionnaire n=56). Continuous variables were presented as means with SD and compared by sex using t-tests. Categorical variables were described by frequencies and compared by sex using χ^2^ tests, with 95% CIs. To evaluate the independent association of different variables on ICH (five to seven ideal metrics), we used univariate and multivariable Poisson regression models adjusting for sex, age groups, site, education level and socioeconomic status and calculated prevalence ratios with 95% CIs. All P values were two-sided and a P<0.05 was considered to be statistically significant. STATA V.10 (StataCorp, College Station, Texas, USA) was used for all analyses.

## Results

A total of 3058 of 3618 (84.5%) of the CRONICAS cohort participants had complete information for analysis. Table 1 presents the baseline sociodemographic characteristics by sex. Mean age of the study population was 55.6 years (SD ±12.7 years) and 51.3% female. Baseline education level and socioeconomic status differed by sex with more females (55.6%) than males (35.6%) having completed only primary school or less (P<0.001). More females (36.1%) were in the poorest tertile of socioeconomic status compared with males (25.8%) (P<0.001).

**Table 1:** Characteristics of the study population overall and by sex (n=3058)

	N	Total population		Female		Male		P value
		N	(%)	N	(%)	N	(%)	
		3058	100	1569	51.30	1488	48.70	
Age group (years)	3057							0.650
35–44		739	24.20	372	23.70	367	24.70	
45–54		786	25.70	417	26.60	369	24.80	
55–64		773	25.30	399	25.40	374	25.10	
≥65		759	24.80	381	24.30	378	24.80	
Site	3058							0.861
Lima		1018	33.30	526	33.50	492	33.00	
Puno urban		503	16.50	257	16.40	246	16.50	
Puno rural		519	17.00	273	17.40	246	16.50	
Tumbes		1018	33.30	513	32.70	505	33.90	
Education	3056							<0.001
Primary or less		1401	45.80	872	55.60	529	35.60	
Secondary		1009	33.00	435	27.70	574	38.60	
Superior		646	21.10	262	16.70	384	25.80	
Wealth index	3058							<0.001
Lowest		950	31.00	566	36.10	384	25.80	
Middle		1052	34.40	527	33.60	525	35.30	
Highest		1056	34.50	476	30.30	580	39.00	

### Prevalence of ICH factors

**Figure 1:**
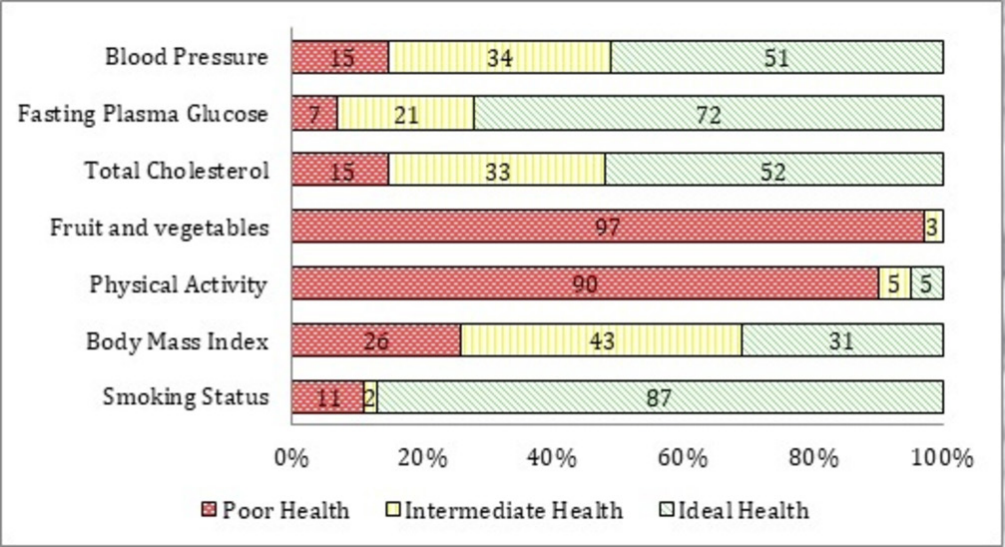
Prevalence estimates for poor, intermediate and ideal cardiovascular health for each of the seven American Heart Association Cardiovascular Health metrics in adults aged ≥35 years (n=3058).

Figure 1 shows the prevalence of poor, intermediate and ideal for each cardiovascular health metric in the total population, weighted for the population size of each study site (Lima, Tumbes, rural and urban Puno). The distribution of number of ICH factors overall and by sex is shown in online supplementary figure 1a.

10.1136/heartjnl-2017-312255.supp1 Supplementary file 1

For ICH factors, ideal fasting plasma glucose was the most prevalent while ideal total cholesterol was the least prevalent. Ideal blood pressure was higher in females than males (63.4% vs 46.5%, respectively) but ideal total cholesterol was lower in females compared with males (48.9% vs 55.1%, respectively). Nearly three-quarters of both females and males had ideal fasting plasma glucose (73.4% vs 74.8%, respectively) (see online supplementary table 2).

10.1136/heartjnl-2017-312255.supp2 Supplementary file 2

### Prevalence of ICH behaviours

The distribution of number of ICH behaviours overall and by sex is shown in online supplementary figure 1b For ICH behaviours, non-smoking was the most prevalent while ideal diet and high physical activity were the least prevalent. Females had higher prevalence of non-smoking than males (95.3% vs 77.1%, respectively). Only one in four females and one in three males had normal BMI. Ideal physical activity levels were low: females 4.0% and males 9.3%. Fruit and vegetable consumption was similar between females and males (7.1% vs 4.9%, respectively) (online supplementary table 2).

10.1136/heartjnl-2017-312255.supp3 Supplementary file 3

### Number of ICH factors and behaviours

Table 2 shows the results for the cardiovascular health metrics by sociodemographic characteristics. No one had all 7 ideal metrics; only 41 (1.3%) had 6 ideal health metrics and 322 (10.5%) had ≤1 ideal health metric. Fewer participants had ICH (5–7 ideal metrics) (12.7%) than had poor cardiovascular health (0–2 ideal metrics) (31.8%). Females, aged 35–44 years, those living in rural Puno and those in the lowest socioeconomic tertile had the highest percentage of ICH (table 2).

**Table 2:** Cardiovascular health metrics by sociodemographic variables

	N	Ideal cardiovascular health (5–7 ideal metrics)	Intermediate cardiovascular health (3–4 ideal metrics)	Poor cardiovascular health (0–2 ideal metrics)	Mean cardiovascular health score (out of 7)
		% (95% CI)	% (95% CI)	% (95% CI)	Mean (95% CI)
Total	3058	12.7 (11.5 to 13.9)	55.6 (53.8 to 57.4)	31.8 (30.1 to 33.4)	3.09 (3.05 to 3.13)
Sex					
Female	1569	13.2 (11.6 to 15.0)	57.7 (55.3 to 60.2)	29.1 (26.9 to 31.4)	3.17 (3.11 to 3.23)
Male	1489	12.1 (10.5 to 13.8)	53.3 (50.8 to 55.9)	34.6 (32.2 to 37)	3.01 (2.94 to 3.07)
Age group (years)					
35–44	739	16.0 (13.5 to 18.8)	60.2 (56.6 to 63.7)	23.8 (20.9 to 27)	3.33 (3.25 to 3.42)
45–54	786	13.7 (11.5 to 16.3)	56.2 (52.7 to 59.7)	30 (26.9 to 33.3)	3.13 (3.04 to 3.22)
55–64	773	8.9 (7.1 to 11.2)	52.9 (49.4 to 56.4)	38.2 (34.8 to 41.6)	2.88 (2.8 to 2.97)
≥65	759	12.1 (10 to 14.6)	53.1 (49.5 to 56.6)	34.8 (31.5 to 38.3)	3.03 (2.94 to 3.11)
Site					
Lima	1018	12.4 (10.5 to 14.6)	55.1 (52.0 to 58.2)	32.5 (29.7 to 35.5)	3.06 (2.98 to 3.13)
Puno—urban	503	13.7 (11 to 17)	63.0 (58.7 to 67.2)	23.3 (19.8 to 27.2)	3.26 (3.16 to 3.37)
Puno—rural	519	23.5 (20 to 27.4)	60.7 (56.4 to 64.8)	15.8 (12.9 to 19.2)	3.64 (3.54 to 3.74)
Tumbes	1018	6.9 (5.5 to 8.6)	49.8 (46.7 to 52.9)	43.3 (40.3 to 46.4)	2.76 (2.69 to 2.83)
Education					
Primary or less	1401	12.3 (10.7 to 14.2)	55.5 (52.8 to 58.1)	32.2 (29.8 to 34.7)	3.10 (3.03 to 3.16)
Secondary	1009	13.6 (11.6 to 15.8)	54.6 (51.5 to 57.7)	31.8 (29 to 34.8)	3.08 (3 to 3.16)
Superior	646	11.9 (9.6 to 14.7)	57.4 (53.6 to 61.2)	30.7 (27.2 to 34.3)	3.10 (3 to 3.19)
Socioeconomic status					
Lowest (poorest)	950	16.8 (14.6 to 19.4)	59.0 (55.8 to 62.0)	24.2 (21.6 to 27)	3.31 (3.23 to 3.39)
Middle	1052	10.8 (9.1 to 12.9)	56.0 (53.0 to 59.0)	33.2 (30.4 to 36.1)	3.05 (2.98 to 3.12)
Highest (richest)	1056	10.7 (9 to 12.7)	52.2 (49.2 to 55.2)	37.1 (34.3 to 40.1)	2.94 (2.86 to 3.01)

The mean ICH score was 3.09 overall, with females, age group 35–44 years, rural Puno, superior education and lowest socioeconomic tertile had the highest mean lifestyle scores (table 2). Males, age groups 55–64 and ≥65 years, semiurban Tumbes, primary education or less and highest socioeconomic status had the highest percentage of poor cardiovascular health (table 2).

Table 3 shows the association between the sociodemographic variables and ICH using univariate and multivariate analyses. Compared with age 35–44 years, the age groups 55–64 years and ≥65 years were less likely to have less ICH after adjusting for sex, site, education and socioeconomic status (P<0.001). Compared with urban Lima, and after adjustment for sex, age, education and socioeconomic status, individuals living in rural Puno were more likely to have ICH, and those living in rural Tumbes less ICH (P<0.001). Compared with those in the lowest tertile of socioeconomic status, those in the middle and highest tertiles were less likely to have ICH (P<0.001) (table 3).

**Table 3:** Association between sociodemographic characteristics and ideal cardiovascular health (5–7 ideal metrics) (n=3058)

	Univariate			Multivariate, adjusted for all other variables		
	PR	95% CI	P value	PR	95% CI	P value
Sex						
Female	Reference			Reference		
Male	0.92	0.75 to 1.12	0.391	0.97	0.79 to 1.19	0.760
Age group (years)						
35–44	Reference			Reference		
45–54	0.86	0.66 to 1.12	0.259	0.85	0.65 to 1.10	0.219
55–64	0.56	0.42 to 0.75	<0.001	0.54	0.40 to 0.74	<0.001
≥65	0.76	0.58 to 1.00	0.048	0.71	0.53 to 0.96	0.028
Site						
Lima	Reference			Reference		
Puno—urban	1.11	0.83 to 1.49	0.492	1.09	0.79 to 1.50	0.594
Puno—rural	1.90	1.48 to 2.44	<0.001	1.61	1.20 to 2.16	0.001
Tumbes	0.56	0.41 to 0.74	<0.001	0.52	0.39 to 0.71	<0.001
Education						
Primary or less	Reference			Reference		
Secondary	1.10	0.88 to 1.38	0.407	1.05	0.81 to 1.35	0.730
Superior	0.97	0.74 to 1.26	0.796	0.96	0.68 to 1.35	0.813
Socioeconomic status						
Lowest (poorest)	Reference			Reference		
Middle	0.64	0.51 to 0.82	<0.001	0.73	0.56 to 0.95	0.02
Highest (richest)	0.64	0.5 to 0.81	<0.001	0.75	0.55 to 1.01	0.06

PR, prevalence ratio.

Of 133 participants with a self-reported history of CVD, fewer with CVD history had ICH (8.2%; 95% CI 3.6% to 13.0%) compared without prior history of CVD (18.4%; 95% CI 17.0% to 19.7%) (P=0.003).

Self-report of ‘very good’ health was low (n=54, 1.8%) in this population; whereas ‘bad’ or ‘very bad’ health was more frequently reported (n=251, 8.2%). Most individuals reported ‘regular’ or ‘good’ health (58.0% and 32.0%, respectively). There were more individuals who reported ‘very good’ or ‘good’ health who had ICH compared with those who did not report ‘very good’ or ‘good’ health (39.2%; 95% CI 35.1% to 43.3% vs 32.6%; 95% CI 30.8% to 34.5%, P=0.003, respectively). Eleven per cent of individuals who reported ‘bad’ or ‘very bad’ health met the ICH metrics (95% CI 8.4% to 13.6%).

### Treatment and control of health factors

Online supplementary table 1 shows medication use and control of blood pressure, TC and fasting plasma glucose. There were 321 (10.5%) participants taking antihypertensive medications, 58 (1.9%) participants taking cholesterol-lowering medications and 94 (3.1%) participants taking glucose-lowering medications in the study population. Of those taking antihypertensive medications, only 77 (24.0%) had their blood pressure controlled. Of those taking cholesterol-lowering medications, 26 (44.8%) had their cholesterol at goal, and 32 (55.2%) had their LDL controlled. Of those taking glucose-lowering medication, 17 (18.1%) had their glucose controlled.

## Discussion

The prevalence of ICH is very low in Peru. Achieving such ideal health status is important as this positive health metric includes factors and behaviours that protect and prevent individuals from developing CVD. In our study, none of the 3058 participants from 4 different geographical regions were found to have all 7 of the ideal health metrics. This is the first study to find substantial differences in ICH metrics among the Peruvian population by sex, age group, site, education level and socioeconomic status. Rural communities, and rural women in particular, are considered to have health inequalities, such as inadequate access to information and limited public investment in health services, housing and education. Poverty affects those in urban and rural areas and around 30% of the population resides at the poverty line, or approximately 9 million people, so this study provides insight into the cardiovascular health of a substantial portion of this population.

The prevalence of having seven ICH metrics is very low globally, ranging from 0.3% to 15% and varies by geographical location, age, sex, ethnicity and education level.^5–10 21 22^ Overall, the mean ICH score in Peru was low at 3.29 (out of 7) and was lower than studies of the US adults aged 20–60 years (3.4–4.7).^9 23^ Rural Tumbes had a mean ICH score of 2.82, which is the lowest score in North and South America (compared with those reported in the literature)— worse than the most unhealthy states in the USA.^9^ Rural Puno and the younger age group (35–44 years) had a higher prevalence than the other sites and age groups of ICH. Prior studies have noted that younger ages are healthier. While only one study looked at a rural population and found 1.0% had all seven ICH metrics and only 7% had ≤1 metric.^24^

In general, ICH factors were more frequent than health behaviours, for example, nearly 75% of participants had normal fasting glucose and over half had normal blood pressure and normal cholesterol. Of those reporting medication use for blood pressure, diabetes or cholesterol, less than half were controlled to goal. This trend has been described previously,^24^ with much lower awareness and control of chronic conditions in low-income and middle-income countries than in high-income countries.

The prevalence of smoking is lower in our study (4.1% in females and 19.9% in males) compared with prior studies in urban areas from Peru, which found age-standardised smoking prevalence of 12.6% for females and 31.1% for males using a similar definition.^12^ Other Latin American populations have even higher prevalence of risk factors, including smoking prevalence of up to 45%,^25 26^ which would further decrease the mean ICH score and result in an even lower prevalence of ICH. The low prevalence of high physical activity and healthy diet were major contributors to the low prevalence of ICH.

The rural communities had the largest difference in percentage of poor cardiovascular health (11.8% in rural Puno vs 41.5% in rural Tumbes) and was the highest socioeconomic tertile (30.8%). The rural sites have different cardiovascular health profiles than those residing in Peru’s capital. In the USA, different prevalence of poor cardiovascular health is seen (6.7% in Colorado to 16.2% in West Virginia), but the variation is lower than this study.^5–9^ Rural Puno is healthier than rural Tumbes even though both populations experience poverty. Rural Puno is located high in the Andean mountains and subsistence farming is common with more traditional diets, consisting mainly of starches (potatoes) and with limited meat; whereas, those living in rural Tumbes have a poor diet with fried meat and fish. Potential factors in Tumbes to account for this difference include hotter climate in Tumbes, different degree of genetic predisposition to developing metabolic derangements and different metabolism due to potential undernourishment during childhood.

Improving the population mean ICH score, by moving from poor to intermediate or intermediate to ideal in any metric, would improve the cardiovascular health to a level similar to that in the worst states in America; however, which specific metric will have the most impact in this population remains unknown. Prior studies suggest that population-based strategies to improve diet and exercise are effective.^27^ The high prevalence of overweight and obesity in this population appears to be associated with these metrics and interventions may decease CVD risk factors.^5 13^ Possible interventions are needed to improve access to and consumption of fruits and vegetables, decreasing obesity, improving the built environment to facilitate exercise, enforcing smoking bans in workplaces and public spaces as well as improving primary healthcare access to deliver preventative health behaviour counselling and perform a health risk assessment.^28^

As with any cross-sectional, population-based study, there may have been selection bias due to the ‘healthy worker effect’ and an overestimation of ICH. The low prevalence of physical activity (which includes both leisure-time and transportation-related) is likely an underestimation of actual physical activity. Future studies could include daily step tracking. Fruit and vegetable intake as a surrogate for diet, which has a protective effect on CVD at high intake^29^ but may overestimate true prevalence of ICH since only 3% met at least 4 of the 5 components of the DASH diet and 25% met this criteria. Medications were self-reported and compliance was not confirmed.

## Conclusions

We report low prevalence rates of healthy lifestyle. The metrics with the lowest prevalence and therefore highest potential room for improvement are health behaviours, including diet quality, physical activity and body weight. This study can serve as a benchmark if ICH and aid in the development of CVD prevention programmes in a resource-limited country with a substantial population living in poverty in rural and urban communities.

**Key messages**

**What is already known about this subject?**

Ideal cardiovascular health is an important predictor of health outcomes and varies by geography and other sociodemographic characteristics.

**What might this study add?**

This study describes the prevalence of seven ideal cardiovascular health factors and behaviours in a resource-constrained setting. No one had all seven ideal metrics; only 41 (1.3%) had 6 ideal health metrics and 322 (10.5%) had ≤1 ideal health metric. Females, aged 35–44 years, those living in rural Puno, and those in the lowest socioeconomic tertile had the highest percentage of ideal cardiovascular health.

**How might this impact on clinical practice?**

The low prevalence of ideal cardiovascular health in this setting is important for clinicians and policy makers to recognise and can serve as a benchmark and aid in the development of cardiovascular disease prevention programmes in a resource-limited country with a substantial population living in poverty in rural and urban communities.
