# Supplementary figures and images for: Low prevalence of ideal cardiovascular health in Peru
Source: Heart. 2018 Jan 11;104(15):1251–6. doi: 10.1136/heartjnl-2017-312255 (PMC6204974; doi:10.1136/heartjnl-2017-312255)

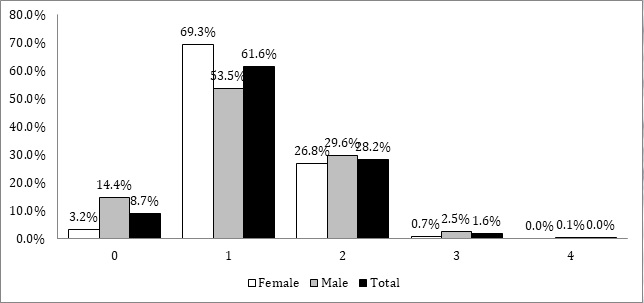

Supplement: Supplementary file 1 [file heartjnl-2017-312255supp001.jpg]

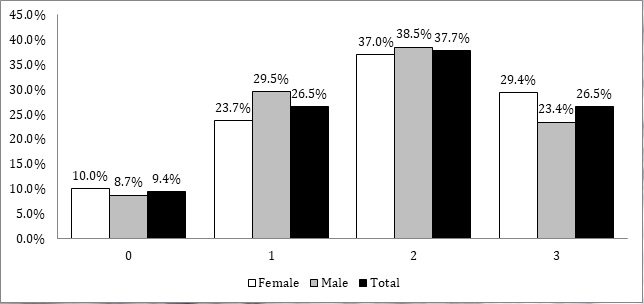

Supplement: Supplementary file 3 [file heartjnl-2017-312255supp003.jpg]
