# Supplementary material for: Low prevalence of ideal cardiovascular health in Peru
Source: Heart. 2018 Jan 11;104(15):1251–6. doi: 10.1136/heartjnl-2017-312255 (PMC6204974; doi:10.1136/heartjnl-2017-312255)
Supplement: Supplementary file 2 [file heartjnl-2017-312255supp002.pdf]

Online Supplemental Material

Online Supplemental table 1. Medication use and control of blood pressure, fasting plasma glucose and total cholesterol as defined by the American Heart Association Ideal Cardiovascular Health metrics.

Variable	N	Poor	Intermediate, not-controlled	Intermediate, controlled*	p-value
**Overall**					
Blood Pressure medication, N (%)	321	128 (39.9%)	116 (36.1%)	77 (24.0%)	<0.001
Glucose-lowering medication, N (%)	94	60 (63.8%)	17 (18.1%)	17 (18.1%)	<0.001
Cholesterol-lowering medication, N (%)	58	12 (20.7%)	20 (34.5%)	26 (44.8%)	0.418
**Female**					
Blood Pressure medication, N (%)	214	84 (39.3%)	76 (35.5%)	54 (25.2%)	<0.001
Glucose-lowering medication, N (%)	54	39 (72.2%)	8 (14.8%)	7 (13.0%)	<0.001
Cholesterol-lowering medication, N (%)	41	10 (24.4%)	14 (34.2%)	17 (41.5%)	0.481
**Male**					
Blood Pressure medication, N (%)	107	44 (41.1%)	40 (37.4%)	23 (21.5%)	<0.001
Glucose-lowering medication, N (%)	40	21 (52.5%)	9 (22.5%)	10 (25.0%)	<0.001
Cholesterol-lowering medication, N (%)	17	2 (11.8%)	6 (35.3%)	9 (52.9%)	0.932

*Intermediate, controlled = measurement for metric falls in the ideal cut-off range but participant was classified as intermediate due to medication use.

Online supplemental table 2. Prevalence estimates for poor, intermediate and ideal cardiovascular health for each of the 7 American Heart Association Ideal Cardiovascular Health metrics in adults aged ≥35 years old by sex (N=3058)*.

	Poor				Intermediate				Ideal			
Variable	Female		Male		Female		Male		Female		Male	
	N	(%)	N	(%)	N	(%)	N	(%)	N	(%)	N	(%)
Blood Pressure	172	-11.00%	239	-16.10%	402	-25.60%	557	-37.40%	995	-63.40%	693	-46.50%
95% CI	9.4%-12.5%		14.2%-17.9%		23.5%-27.8%		35.0-39.9%		61.0-65.8%		44.0-49.1%	
Fasting Plasma Glucose	103	-6.60%	76	-5.10%	314	-20%	298	-20%	1152	-73.40%	1115	-74.90%
95% CI	5.3%-7.8%		4.0%-6.2%		18.0%-22.0%		18.0%-22.1%		71.2-75.6%		72.7-77.1%	
Total Cholesterol	292	-18.60%	195	-13.10%	510	-32.50%	473	-31.80%	767	-48.90%	821	-55.10%
95% CI	16.7%-20.5%		11.4%-14.8%		30.2%-34.8%		29.4%-34.1%		46.4%-51.4%		52.6%-57.7%	
Healthy Diet Score	598	38.11%	698	46.88%	958	61.06%	776	52.12%	13	0.83%	15	1.01%
95% CI	35.7%-40.5%		44.3%-49.4%		58.6%-63.5%		49.6%-54.7%		0.4% - 1.3%		0.5% - 1.5%	
Fruit and vegetables	1458	92.93%	1416	95.10%	n/a				111	7.07%	73	4.90%
95% CI	91.7% - 94.2%		94.0% - 96.2%						5.8% - 8.3%		3.8% - 6.0%	
Physical Activity	1458	-92.90%	1219	-81.90%	49	-3.10%	132	-8.90%	62	-4.00%	138	-9.30%
95% CI	91.7%-94.2%		79.9%-83.8%		2.3%-4.0%		7.4%-10.3%		3.0%-4.9%		7.8%-10.7%	
Body Mass Index	545	-34.70%	284	-19.10%	632	-40.30%	715	-48.00%	392	-25.00%	490	-32.90%
95% CI	32.4%-37.1%		17.1%-21.1%		37.9%-42.7%		45.5%-50.6%		22.8%-27.1%		30.5%-35.3%	
Current Smoking	64	-4.10%	296	-19.90%	10	-0.60%	45	-3.00%	1495	-95.30%	1148	-77.10%
95% CI	3.1%-5.1%		17.9%-21.9%		0.2%-1.0%		2.2%-3.9%		94.2%-96.3%		75.0%-79.2%	

*Reference: (22) | Abbreviations: CI, confidence interval.
